# Preliminary Deformational Studies on a Finite Element Model of the Nasal Septum Reveals Key Areas for Septal Realignment and Reconstruction

**DOI:** 10.1155/2013/250274

**Published:** 2013-04-15

**Authors:** Kyrin Liong, Shu Jin Lee, Heow Pueh Lee

**Affiliations:** ^1^Department of Mechanical Engineering, National University of Singapore, Singapore 117576; ^2^Division of Plastic, Reconstructive and Aesthetic Surgery, National University Hospital, Singapore 119074

## Abstract

*Background*. With the current lack of clinically relevant classification methods of septal deviation, computer-generated models are important, as septal cartilage is indistinguishable on current imaging methods, making preoperative planning difficult. *Methods*. Three-dimensional models of the septum were created from a CT scan, and incremental forces were applied. *Results*. Regardless of the force direction, with increasing force, the septum first tilts (type I) and then crumples into a C shape (type II) and finally into an S shape (type III). In type I, it is important to address the dislocation in the vomer-ethmoid cartilage junction and vomerine groove, where stress is concentrated. In types II and III, there is intrinsic fracture and shortening of the nasal septum, which may be dislocated off the anterior nasal spine. Surgery aims to relieve the posterior buckling and dislocation, with realignment of the septum to the ANS and possible spreader grafts to buttress the fracture sites. *Conclusion*. By identifying clinically observable septal deviations and the areas of stress concentration and dislocation, a straighter, more stable septum may be achieved.

## 1. Introduction

Nasal septal deviation is a common nasal deformity. It can be a congenital disorder or a consequence of nasal trauma. Deviation of the bony or cartilaginous component of the nasal septum from the midline leads to its deviation. This results in external nasal deformity, internal nasal obstruction due to nasal airway constriction, or a combination [1–3].

Presently, septal deviation classification has largely been descriptive, based on nasal septal geometry and relationships between the bony and cartilaginous septa [4–7]. Jang et al. [6] presented a simplified classification of nasal deviation and the associated treatment outcome into five types based on the orientation of the bony pyramid and the cartilaginous vault. Jin et al. [7] presented a four-category classification of septal deviation based on the morphology, site, severity, and its influence on the external nose. Buyukertan et al. [4] reported a morphometric study of nasal septal deviation by separating the nasal septum into 10 segments. They concluded that the system would constitute a new, objective, simple, and practical classification system. I. Baumann and H. Baumann [8] argued that the existing nomenclatures of septal deviation only dealt with nasal septum deformation exclusively and were rarely used in routine clinical work. They instead presented a method for the classification of septal deviations based upon the anatomical structures of the nasal septum and common clinical concepts. However, the most observable nasal septal deviation classification system was proposed by Rohrich et al. [9]. Therefore, for simplicity, nasal septal deviations will be classified according to that proposed by Rohrich et al. [9].

In order to improve the clinical outcome of septoplasty, a greater understanding of the etiopathogenesis of nasal septal deviation is necessary. This requires a basic understanding of the biomechanics of its formation. We aim to apply incremental force to a computer-generated septal model using structural modal analysis, which has also been utilized by Laura et al. [10], who previously described a simple method for determining the fundamental mode of a vibrating ulna to approximate its dynamic response. The objective of this study is to identify areas of high-stress and septal deformation patterns. Clinically, this may assist surgeons in the delineation of key areas for septal realignment and reconstruction.

## 2. Materials and Methods

### 2.1. Generation of Cranial Computed Tomography (CT) Scan and Finite Element Models

**Figure 1:**
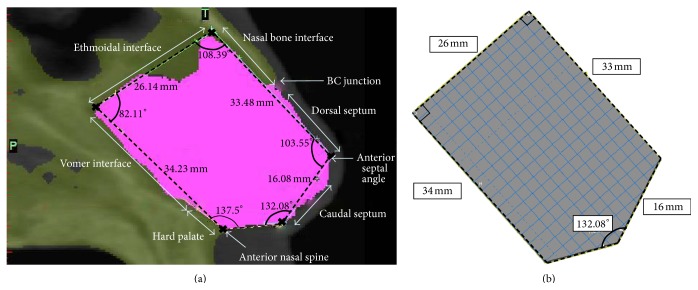
(a) Sagittal CT scan of the nasal septum with the indicated anatomical features and their measurements. (b) Idealized cartilaginous septum model created with the indicated measurements.

Cranial CT scans were obtained from a patient who possessed normal features—a straight nasal septum, normal occlusion and a perceivably symmetric face (Figure 1(a)). This study was performed in accordance with the guidelines of the institutional review board (IRB) and conforms to the Helsinki's Declaration. The patient had not previously undergone septoplasty or rhinoplasty, nor subject to nasal injury. Superposition of the CT images to create a three-dimensional (3D) model was conducted with Mimics software (Materialise Technologies, Leuven, Belgium). An idealized model (Figure 1(b)) and a patient-specific finite element model were generated for the study. 

From the CT scans, we observed that the cartilaginous nasal septum was present in five slices. We chose to base the idealized model on the middle slice (Figure 1(a)) and measured the significant features of the nasal septum. We then utilized these measurements to create an idealized model (Figure 1(b)) in the Finite Element Analysis software, ABAQUS (Dassault Systèmes Technologies, Providence, RI, United States of America (USA)), where the idealized model was, subsequently, meshed. We recognize the thickness variation present in the septum. However, to simplify analyses and gain an estimate of nasal deformation, models were prescribed with a uniform thickness of 2 mm [11], which is an approximate average septum thickness, as reported previously [11–13]. To ensure mesh accuracy, convergence studies were carried out on the model.

To create a more realistic representation of the septum, which incorporated thickness variation, a 3D patient-specific model was created from the same CT scan utilizing Mimics software (Materialise Technologies, Leuven, Belgium) and meshed with Hypermesh (Altair HyperWorks, Troy, MI).

### 2.2. Material Properties

Cartilage exhibits a “nonhomogenous, anisotropic, nonlinear, viscoelastic behaviour” [14]. For deformations below 20% [14], however, no significant changes occur within the cartilage, and it is therefore sufficiently accurate to model cartilage as a homogenous, linearly elastic material in our analyses [15]. Mau et al. [11] utilized a similar homogenous, linear elastic material property to simulate septal L-strut deformation.

To define the linear elastic model of the cartilage, the Young's modulus, *E*, and Poisson's ratio, *v*, are required. However, the tensile and compressive Young's moduli are vastly different due to the structure of cartilage. According to Lee et al. [16], the tensile modulus ranges from 2.62 MPa to 10.6 MPa, the compressive modulus ranges from 0.40 MPa to 0.83 MPa, and the Poisson's ratio ranges from 0.26 to 0.38.

Specimen density is also required in the analysis. Cartilage is approximately 75% water, while the other 25% consists mainly of type-two collagen fibrils and proteoglycan molecules [17]. The density of water is 1000 kg/m^3^, while the other components are highly dense structures. Therefore, the density of cartilage was estimated to be 2000 kg/m^3^. 

As the relative displacement within the septum is the main area of concern in this analysis, and since material properties affect the absolute and not the relative displacement of the septum, the average values of the elastic modulus and Poisson's ratio and an estimated value of the density were used. The elastic modulus was assigned a value of 5 MPa, Poisson's ratio was 0.32, and the density was 2000 kg/m^3^.

### 2.3. Boundary Conditions

#### 2.3.1. Bony Interfaces

As the bony interfaces with the nasal septum—ethmoidal, vomer, hard palate, and nasal bone interfaces (Figure 1(a))—are much stiffer than the septal cartilage, most of any applied force will be absorbed by the cartilaginous septum, leaving the bony septum uninjured [18]. It is therefore reasonable to assume these interfaces as rigidly fixed [15].

The nasal bone length overlapping the cartilaginous septum may affect the degree of nasal deformation and normally ranges from 3 to 15 mm [19]. However, to simplify analyses, a candidate that displayed a length that fell within this range—in this case, 14 mm—was considered, so that a typical deformation pattern could be observed. 

#### 2.3.2. Nasal Tip

In vivo, the nasal tip lies anterior to the anterior septal angle (ASA) where the lower lateral cartilages (LLCs) meet, although this may vary. However, due to the small distance between the ASA and the nasal tip, and for simplification in this analysis, the ASA was assumed to be the nasal tip. 

According to Lee et al. [16], the nasal tip cartilages may be thought of as a spring and a cantilever, as they exhibit deformation recoil and elasticity. A cantilever is a result of the unequal stability in the tripod formed by the medial crura and paired LLCs, and a spring results from the LLCs, which produce an upward force that is in the form of stored elastic potential energy [16]. Therefore, the nasal tip may be modeled as spring supported.

The spring-stiffness constant, *k*, may be defined by (1) [20], and a spring-stiffness constant of 20 kN/m is applied in the three orthogonal axes.

(1) $$\begin{array}{c} k=\frac{E{\left(\text{width}\times \text{height}\right)}^{3}}{4{\left(\text{length}\right)}^{3}}. \end{array}$$

**Figure 2:**
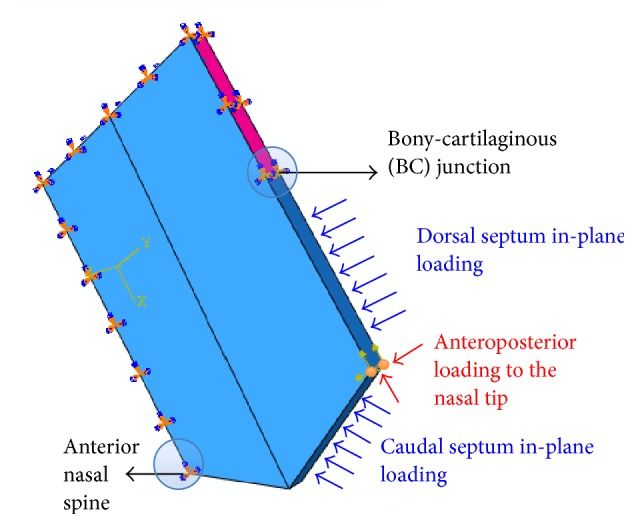
Three-dimensional idealized finite element model of the nasal septum with anteroposterior loading to the nasal tip, dorsal and caudal septa in-plane loading, and indicated anatomical features.

A free nasal tip was prescribed as a preliminary step. A spring-supported nasal tip boundary condition, where the spring was connected between the two orange points on the nasal tip (Figure 2), was then applied to compare the effect of different boundary conditions. The dorsal and caudal septa were prescribed a free boundary condition. 

### 2.4. Loading Conditions

As “frontal force to the septum causes damage ranging from simple fracture of the nasal bones to severe flattening of the nasal bones and the septum” [21], two forms of frontal loading were applied—anteroposterior and dorsal and caudal septa in-plane loading. The force and pressure applied are estimates and are inconsequential to the relative displacements of the septum. As the present intention is to determine the Eigen modes, or the most likely deformed patterns of the septum, only the possible in-plane loading which will affect the resulting Eigen modes will be considered.

In the case of anteroposterior loading (Figure 2), a couple of forces of 1N each in both the vertical and horizontal axes were applied to the nasal tip to simulate a direct frontal punch at an angle such that the forces on the nasal tip are the most significant.

In the case of dorsal and caudal septa in-plane loading (Figure 2), a uniform pressure of 2000 Pa was applied to both the dorsal and caudal septa. This was to simulate a frontal punch at an angle such that both the dorsal and caudal septa components are equally significant.

### 2.5. Eigen Modes and Finite Element Simulation

Every object, including the septum, has a set of Eigen modes, depending on its structure and composition [22]. In each mode, all parts of the system vibrate with the same distinct frequency, which is referred to as the system's Eigen value at that mode. The lowest frequency is referred to as the fundamental frequency [23]. Since lower modes have lower frequencies and energies, they are more likely to occur. Hence, only the first 10 modes of the nasal septum were analyzed.

ABAQUS (Dassault Systèmes Technologies, Providence, RI) was used to obtain the Eigen mode shapes of the septum under the various loading conditions. A general, static step is created, in which one of the two loading conditions is applied. Thereafter, a linear perturbation, frequency step is created, in which the natural frequency and the corresponding mode shape will be extracted.

## 3. Results

**Figure 3:**
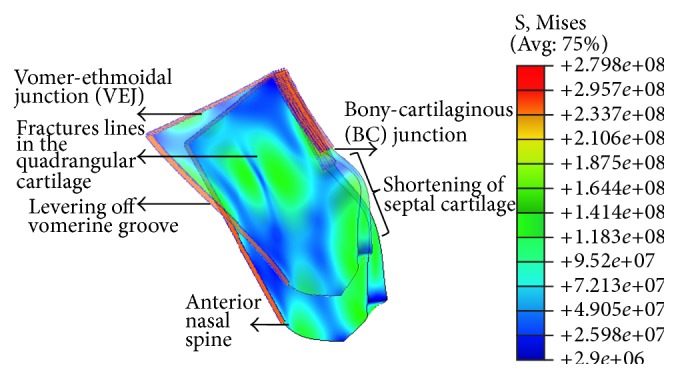
Intrinsic points of fatigue in the idealized cartilaginous septum model and the resultant shortening of the cartilage and levering off the vomerine groove. Note the stresses shown are Von Mises stresses. The ghost image that illustrates the shortening and levering of the septum is for illustrative purposes only.

The patterns of nasal septal deviation were similar to those described by Rohrich et al. [9]. In our study, the deviation patterns were therefore classified into three groups, each with its specific sites of high stress and dislocation and possible surgical corrective procedures (Table 1). Through observation of all deformation patterns, we were also able to identify the intrinsic points of fatigue within the cartilaginous septum—the BC junction, anterior nasal spine (ANS), vomer-ethmoidal cartilage junction (VEJ), and a single or couple of cracks in the quadrangular cartilage that lead to C-shaped and S-shaped nasal deformations, respectively. These points could lead to the septum levering off the vomerine groove and, in the latter two cases, a shortening of the septum (Figure 3).

**Figure 4:**
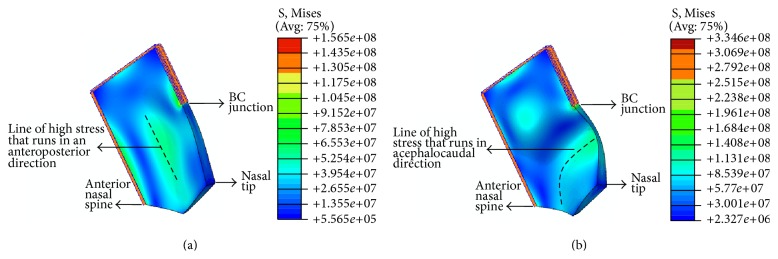
(a) Idealized nasal septum model with an unrestricted nasal tip in mode 3, displaying a C-shaped septum, with a line of high stress running through the anteroposterior direction. (b) Idealized nasal septum model with an unrestricted nasal tip in mode 4, displaying a C-shaped septum, with a line of high stress running through the cephalocaudal direction. Note that the stresses shown are Von Mises stresses.

**Figure 5:**
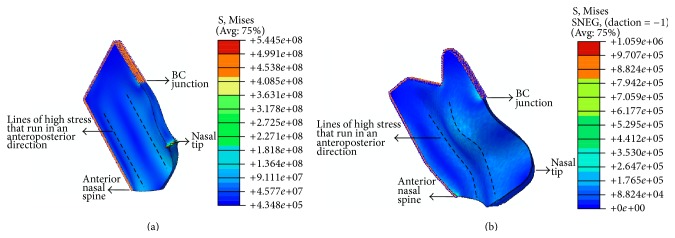
(a) Idealized nasal septum model with a spring-supported nasal tip in mode 6, displaying an S-shaped septum, with two lines of high stress running in the anteroposterior direction. (b) Patient-specific nasal septum model with a spring-supported nasal tip in mode 6, displaying an S-shaped septum, with relatively greater displacements than the corresponding idealized model. Note that the stresses shown are Von Mises stresses.

For an idealized model, the slanted (Figure 4(a)), C-shaped (Figure 4(b)), and S-shaped (Figure 5(a)) deviation patterns were all observed (Table 2). In some modes, the system vibrates in-plane and therefore lacks a resultant deformation shape. In such cases, a dash is indicated. However, due to the lack of restriction on the nasal tip, it moves relatively freely, which may not represent in the vivo conditions. 

In the following idealized model, the nasal tip is now constrained by a spring. While displaying similar patterns of deviation with a free nasal tip model, the spring-supported nasal tip model exhibits decreased displacement due to its prescribed restriction.

A patient-specific model was then analysed. By observation of the previous idealized models, it became apparent that the two forms of loading produced almost identical results. Therefore, only anteroposterior loading was prescribed to this model. The patient-specific model exhibited similar deformation patterns as the idealized nasal septal models (Table 2 and Figure 5).

## 4. Discussion

### 4.1. General Findings

The nasal septum is of utmost importance in the support of the “distal nose and for the maintenance of the bilateral nasal airway” [24]. A straight septum exists where there is force equilibrium [25], which may be disturbed in fracture, resulting in warping of the septal cartilage [18, 26]. Depending on the sustained trauma, the septum may deform in a myriad of patterns. Presently, however, studies have reported that septal deformation patterns may be categorized in a number of broad categories, regardless of the trauma and/or injuries sustained. Guyuron et al. [5], Rohrich et al. [9], and Rhee et al. [24] categorized nasal deformities broadly into a septal tilt, anteroposterior or cephalocaudal C-shaped and an S- or reverse-S-shaped deformities. Unfortunately, these studies have not correlated these deviation patterns with degrees of force. Through the correlation of septum deformation patterns with increasing degrees of force, as well as with areas of dislocation and fracture, preoperative planning and septoplasty may be improved. The prompt identification and management of septal fractures are necessary to avoid nasal obstruction and posttraumatic septal deformity [24]. 

In our analyses, we were able to identify clinically observable nasal septal deviations and the aforementioned high-stress areas that would require stress-relief and the possible dislocation sites (Figure 3).

### 4.2. Prevalence of Modal Shapes and Concentrated Stress Zones in Idealized Models

We observed that regardless of force direction, with increasing force, the septum first tilts (type I) and then crumples into a C-shape (type II) and finally into an S-shape (type III). This was observed through the prevalence of the tilted septum in lower modes, while the “C-shaped” followed by the “S-shaped” deformation shapes occurring in relatively higher modes, respectively. As lower modes require less energy to manifest, they occur more frequently. Therefore, the lower the mode in which the deviation pattern is observed, the smaller the force required to cause this deformation, and consequently, the greater the probability of observing this pattern. Our findings are in agreement with clinical experience. According to Guyuron et al. [5], in a sample size of 93 patients who had undergone primary septoplasty, 40% had a septal tilt, 32% had a C-shape anteroposterior septum, 4% had a C-shape cephalocaudal septum, 9% had an S-shape anteroposterior septum, and 1% had an S-shape cephalocaudal septum.

In type I, when a tilted septum is observed, the highest stress concentration occurs at the BC junction and ANS. These high-stress areas were also reported by Lee et al. [21]. This suggests that with a low to moderate force, the septum dislocates en-mass from the midline vomerine groove (Figure 3) and levers off the BC junction to a tilted position. This may be observed on CT and MRI scans, and naso-endoscopy, where posterior buckling is frequently observed at the VEJ. Through submucous resection, the septum may be repositioned onto the groove [5], with prior resection of the cartilage tongue in the nasal floor. The septum may then be reset to the ANS (Table 1).

With a higher moderate force, a “C-shaped” deformation is likely due to a central line of stress in the septum, bending it into two pieces. The line of high stress may run through the anteroposterior (Figure 4(a)) or cephalocaudal (Figure 4(b)) directions. We propose that with significant loading, intrinsic septal fractures occur by breaking the cartilaginous septum into two, leading to the clinical morphology of a C-shaped nose and shortening of the septum. In addition, the septum will be displaced off its vomerine groove and/or the ANS and will likely buckle at the VEJ (Figure 3). This is clinically significant as it cannot be observed on CT or MRI scans due to the invisibility of the septum in such modalities [27]. Hence, a clinical observation of a C-shaped septum may be the only indication. In addition to the corrective procedures mentioned previously, spreader grafts may be required on both sides of the septum, to assist in its straightening by providing the necessary nasal support that was relinquished when the septum fractured, thereby allowing the septum time to heal (Table 1). 

With a force of higher magnitude, an “S-shaped” deformation may result due to multiple lines of stress leading to a septal concertina and shortening of the septum into a minimum of three overlapping pieces [28]. This is due to two lines of stress running in the anteroposterior direction (Figure 5). In addition to being shortened, the septum might be displaced from the vomerine groove and ANS (Figure 3). As with a C-shaped deformed septum, a clinical observation is its sole indication [27]. In addition to the aforementioned corrective procedures, longer spreader grafts will be required to brace both deformed sites to support the septum [5] and allow it to heal (Table 1).

Therefore, regardless of the deviation pattern, by relieving stress in these specific strips of concavity, in combination with the aforementioned surgical procedures, we propose that a more stable, straight septum may be achieved.

### 4.3. Comparison of Various Loading and Boundary Conditions

Despite different loading conditions, the nasal septum deviates in a relatively constant pattern of a septal tilt, C- and S-shaped deviations with insignificant differences between the resultant modal shapes. 

The free nasal tip and spring-supported nasal tip models responded differently to the loading conditions, specifically in mode three. A septal tilt is observed in the free nasal tip model, while a C-shaped deformation is observed in the latter model. As the C-shape deviation is noted to occur with higher energy and septal tilt deviation with lower energy, this finding suggests that the spring of the LLCs acts to insulate and constrain the nasal tip and septum against deformation. The protective interrelationship of the LLCs to the nasal septum should be preserved during surgery.

### 4.4. Comparison of the Patient-Specific and Idealized Models

The prevalence of modal shapes in patient-specific and idealized septal models, subject to frontal point-loading, is almost identical (Table 1). Slight deviations, such as those in mode three, are expected, due to the difference in shape between the models. Despite the patient-specific model exhibiting greater relative movement than the idealized model, this difference is insignificant as the basic modal shape remains (Figure 5). The similarities observed between these two models are a testament to the accuracy of the idealized model.

### 4.5. Limitations of the Study

It is imperative to note that nasal septal deviations are secondary to the bony vault and cartilaginous changes. For the purpose of this study, the focus is on septal cartilage deformation patterns. Future research aims to combine the study of the deformations of the bony and cartilaginous septa. Due to the inherent collagen fibrils and the consequent anisotropy within the cartilaginous septum, we recognize that the prescription of a linearly elastic material model to the nasal septum material properties may not be fully representative of in vivo cartilage. In spite of this, an understanding of the relative displacement that occurs within the different models in different Eigen modes remains beneficial in aiding surgeons to correct a deviated nasal septum. No physical model was mechanically tested to validate the computational model in this preliminary study, which means that absolute stresses and relative stress patterns should be considered cautiously. Such an experimental validation study would typically make use of strain gages, but also infrared thermography, and global stiffness measurements [29, 30].

## 5. Conclusion

The purpose of this study was to gain a greater understanding of the septal deformation biomechanics. We found that despite different loading directions, the septum deformed consistently into only three shapes—a tilted position, a C-shaped septum, and an S-shaped septum. These patterns are in agreement with clinical observations of septal deformation patterns. The tilted septum is seen with the least force, C shape with moderate force, and S shape with high force. This suggests an intrinsic fracture of the septum into increased number of overlapping fragments with escalating force. Clinically, this is important information that provides insight into predictable patterns of internal septal fractures that need to be realigned and reconstructed to create a straight septum. 

## Figures and Tables

**Table 1:** Classification of septum deviation pattern based on sites of dislocation and possible surgical corrective procedures.

Type	Septum deviation pattern	Sites of dislocation	Surgical corrective procedures
I	Tilted in one piece	(i) Vomer-ethmoidal-cartilaginous (VEC) buckling (ii) Lower edge of septum dislocated off vomerine groove (iii) ANS attachment may be intact	(i) Submucous resection (ii) ±Septal reset to ANS (iii) ±Septal extension grafts for tip support

II	C-shaped	(i) Vomer-ethmoidal-cartilaginous (VEC) buckling (ii) Lower edge of septum dislocated off vomerine groove (iii) ANS attachment may be intact (iv) Septal fracture in a single site	(i) Septal reset to the ANS (ii) Submucous resection (iii) Inclusion of spreader grafts to buttress the septal fracture (iv) Possible septal extension grafts for vertical septal fracture

III	S-shaped	(i) Vomer-ethmoidal-cartilaginous (VEC) buckling (ii) Lower edge of septum dislocated off vomerine groove (iii) ANS attachment may be intact (iv) Septal fracture possibly in two sites, forming a septal concertina	(i) Septal reset to the ANS (ii) Submucous resection (iii) Inclusion of spreader grafts to buttress the septal fracture (iv) Inclusion of septal extension grafts to restore the support that is lost with septal shortening

**Table 2:** Resultant modal shapes for idealized septum models with an unrestricted and spring-supported nasal tip and in the patient-specific nasal septum model with a spring-supported nasal tip.

Idealized nasal septum models						Patient-specific nasal septum model	
Unrestricted nasal tip			Spring-supported nasal tip			Spring-supported nasal tip	
Mode	Anteroposterior loading	Dorsal and caudal in-plane loading	Mode	Anteroposterior loading	Dorsal and caudal in-plane loading	Mode	Frontal point loading
1	I	I	1	I	I	1	I
2	II	II	2	II	II	2	II
3	II	II	3	I	I	3	II
4	II	II	4	II	II	4	II
5	II	II	5	II	II	5	II
6	III	III	6	III	III	6	III
7	—	—	7	—	—	7	II
8	III	III	8	III	III	8	—
9	II	II	9	II	II	9	III
10	III	III	10	III	III	10	III
